# Differential Gene Expression Profile of Renin-Angiotensin System in the Left Atrium in Mitral Regurgitation Patients

**DOI:** 10.1155/2018/6924608

**Published:** 2018-11-18

**Authors:** Wen-Hao Liu, Yen-Nan Fang, Chia-Chen Wu, Mien-Cheng Chen, Jen-Ping Chang, Yu-Sheng Lin, Kuo-Li Pan, Wan-Chun Ho, Tzu-Hao Chang, Yao-Kuang Huang, Chih-Yuan Fang, Chien-Jen Chen, Wei-Chieh Lee

**Affiliations:** ^1^Division of Cardiology, Department of Internal Medicine, Kaohsiung Chang Gung Memorial Hospital, Chang Gung University College of Medicine, Kaohsiung, Taiwan; ^2^Division of Cardiovascular Surgery, Kaohsiung Chang Gung Memorial Hospital, Kaohsiung, Taiwan; ^3^Division of Cardiology, Chang Gung Memorial Hospital, Chiayi, Taiwan; ^4^Graduate Institute of Biomedical Informatics, Taipei Medical University, Taipei, Taiwan; ^5^Department of Thoracic and Cardiovascular Surgery, Chang Gung Memorial Hospital, Chiayi, Taiwan

## Abstract

**Background:**

Left atrial enlargement is a mortality and heart failure risk factor in primary mitral regurgitation (MR) patients. Pig models of MR have shown differential expression of genes linked to the renin-angiotensin system. Therefore, the aim of this study was to investigate the key genes of the renin-angiotensin that are expressed differentially in the left atrial myocardium in MR patients.

**Methods:**

Quantitative RT-PCR was used to compare gene expression in the renin-angiotensin system in the left atrium in MR patients, aortic valve disease patients, and normal subjects.

**Results:**

Plasma angiotensin II concentrations did not significantly differ between MR patients and aortic valve disease patients (*P* = 0.582). Compared to normal controls, however, MR patients had significantly downregulated expressions of angiotensin-converting enzyme, angiotensin I converting enzyme 2, type 1 angiotensin II receptor, glutamyl aminopeptidase, angiotensinogen, cathepsin A (*CTSA*), thimet oligopeptidase 1, neurolysin, alanyl aminopeptidase, cathepsin G, leucyl/cystinyl aminopeptidase (*LNPEP*), neprilysin, and carboxypeptidase A3 in the left atrium. The MR patients also had significantly upregulated expressions of *MAS1* oncogene (*MAS1*) and mineralocorticoid receptor compared to normal controls. Additionally, in comparison with aortic valve disease patients, MR patients had significantly downregulated *CTSA* and *LNPEP* expression and significantly upregulated *MAS1* expression in the left atrium.

**Conclusions:**

Expressions of genes in the renin-angiotensin system, especially *CTSA*, *LNPEP*, and *MAS1*, in the left atrium in MR patients significantly differed from expressions of these genes in aortic valve disease patients and normal controls. Notably, differences in expression were independent of circulating angiotensin II levels. The results of this study provide a rationale for pharmacological therapies or posttranslational regulation therapies targeting genes expressed differentially in the renin-angiotensin system to remedy structural remodeling associated with atrial enlargement and heart failure progression in patients with MR.

## 1. Introduction

Mitral regurgitation (MR) is the second most prevalent valvular heart disease after aortic valve stenosis [1] and a major cause of heart failure (HF). Even under medical treatment, annual mortality rates approximate 3% in patients with moderate primary MR and 6% in patients with severe primary MR [2]. Left atrial enlargement is also an important risk factor for mortality, HF, and cardiac events in primary MR patients under regular medical management [3]. Notably, atrial myocardial stretch with left atrial enlargement results from volume overload caused by an MR with a blood jet into the left atrium. In MR patients with HF, structural remodeling associated with atrial enlargement may also occur in the left atrial myocardium [4, 5]. A previous study in adult rats also revealed that myocyte stretching that mimics volume overload can induce angiotensin II synthesis and myocyte apoptosis, which could then be abolished by angiotensin II type I receptor blocker [6]. Interestingly, a pig model of MR in our previous study revealed differential expression of genes related to structural remodeling of the left atrium [7]. Analyses of gene ontology and pathway enrichment also reveal differential expression of renin-angiotensin system genes in the KEGG pathway in the left atrial myocardium of MR pigs [7]. Moreover, some of the genes expressed differentially in the left atrial myocardium could be regulated by angiotensin II type I receptor blocker [7]. However, the key genes of the renin-angiotensin system that are expressed differentially in the left atrial myocardium of MR patients have never been explored. Therefore, the aim of this study was to determine what key genes of the renin-angiotensin system are expressed differentially in the left atrium in severe MR patients with HF compared to normal controls. Since the left atrium is smaller in aortic valve disease patients compared to MR patients, the left atrial myocardium specimens from severe aortic valve disease patients with HF were also used as a separate cohort for gene analyses. The information regarding renin-angiotensin system and the KEGG pathway was obtained online from GenomeNet Database Resources (https://www.genome.jp/kegg-bin/show_pathway?hsa04614). This study revealed that several key element genes of the renin-angiotensin system were differentially expressed in the left atrium in MR patients in comparison with aortic valve disease patients and normal controls. Notably, the differential expressions were independent of circulating angiotensin II levels.

## 2. Materials and Methods

### 2.1. Ethics Statement

The study protocol conformed to the ethical guidelines of the 1975 Declaration of Helsinki and was approved by the Institutional Review Committee for Human Research of Chang Gung Memorial Hospital (102-2219C). Written informed consent was obtained from each study patient.

### 2.2. Patient Population

This study enrolled 18 severe nonischemic MR patients with HF in sinus rhythm (age: 57 ± 11 years), 12 patients with severe degenerative aortic valve disease and HF in sinus rhythm (age: 60 ± 12 years; aortic stenosis in 5, aortic regurgitation in 5, combined aortic stenoregurgitation in 2), and 16 control subjects without valve disease and HF. Exclusion criteria included previous myocardial infarction, febrile disorder, infectious or inflammatory disease, autoimmune disease, malignancy, acute and/or chronic viral hepatitis, and current use of immunosuppressive drugs.

Eleven left atrial tissue samples from normal adults were purchased for use as normal controls. Of these, six tissue samples were used for gene studies (24-year-old Caucasian male, 27-year-old Caucasian male, 30-year-old Asian male, 60-year-old Caucasian female, 76-year-old Caucasian female, and 77-year-old Caucasian male; BioChain, Newark, CA, USA). Five tissue samples were used for measuring tissue angiotensin II and angiotensin 1~7 concentrations. Of these, one sample (35-year-old Caucasian female) was obtained from G-Biosciences (St Louis, MO, USA), and four samples (49-year-old African American male, 60-year-old Caucasian female, 62-year-old Caucasian female, and 77-year-old Caucasian male) were obtained from BioChain (Newark, CA, USA).

### 2.3. Specimen Storage

During surgery, small specimens of atrial tissue were collected from the left atrial free wall of patients with MR and aortic valve disease. Excised atrial tissues were immediately frozen in liquid nitrogen and stored at −80°C for subsequent analyses.

Blood samples collected from MR patients, aortic valve disease patients, and control subjects without valve disease and HF were stored in tubes containing EDTA. The blood samples were centrifuged at 3000 rpm for 10 minutes at 4°C, aliquoted into Eppendorf tubes, and stored at −80°C.

### 2.4. Measurement of Plasma Angiotensin II and Angiotensin 1~7 Concentrations

Plasma angiotensin II concentration was measured with an enzyme immunoassay (EIA) kit (Cayman Chemical, Ann Arbor, USA) according to the manufacturer instructions. The EIA kit includes angiotensin standard, antiangiotensin II IgG tracer, glutaraldehyde, borane trimethylamine, Ellman reagent, assay, and wash buffers. The standard curve range was 0.98–125 pg/mL. An aliquot of plasma was assayed, and all samples were tested in duplicate. The EIA plate was read at 405 nm with an auto plate reader (*μ*Quant, Bio-TEK, Vermont, USA). The mean intra-assay coefficient of variances of angiotensin II was 4.9%.

Plasma angiotensin 1~7 concentrations were measured with a human angiotensin 1~7 ELISA kit (MyBioSource, San Diego, USA) according to the manufacturer instructions.

### 2.5. Measurement of Tissue Angiotensin II and Angiotensin 1~7 Concentrations in the Left Atrium

To determine angiotensin II and angiotensin 1~7 concentrations, human left atrial tissues were dissected and lysed by sonication with RIPA buffer (Cell Signaling, MA, USA) and supplemented with 1% protease inhibitors. The lysates were incubated on ice for 30 minutes and then cleared with centrifugation. The supernatants were used to measure angiotensin II and angiotensin 1~7 concentrations in the left atrium with a human angiotensin II EIA kit (Cayman Chemical, Ann Arbor, USA) and a human angiotensin 1~7 ELISA kit (MyBioSource, San Diego, USA) according to the manufacturer instructions.

### 2.6. Quantitative Determination of RNA by Real-Time RT-PCR

The RNAs were extracted from the left atrial myocardial tissue using a RiboPure™ kit (Ambion, NY, USA) according to the manufacturer protocol and then reverse transcribed to cDNA using the high-capacity cDNA reverse transcription kit (Applied Biosystems, Foster City, CA). Real-time quantitative PCR was performed using TaqMan chemistry on a 7500 Fast Real-Time PCR System (Applied Biosystems). TaqMan primers and probe mixtures were also purchased from Applied Biosystems.  Table 1 shows the TaqMan real-time PCR assay identifications. The results were normalized against GAPDH gene expression (endogenous control). Quantitative RT-PCR values were presented in △Cq units.

### 2.7. Statistical Analysis

Data were presented as mean ± SD (baseline characteristics) or SEM (plasma and tissue angiotensin II and angiotensin 1~7 concentrations and gene expressions). Categorical variables (excluding New York Heart Association functional class) between MR patients and aortic valve disease patients were compared using Fisher exact tests. Categorical variables among MR patients, aortic valve disease patients, and control subjects were compared using chi-square test. Chi-square test was also used to compare New York Heart Association functional class between MR patients and aortic valve disease patients. Continuous variables among three groups were analyzed by Kruskal-Wallis test, and continuous variables between two groups were analyzed by Mann-Whitney test. Covariates were adjusted according to analysis of covariance results. Statistical analysis was performed using commercial statistical software (SPSS for Windows, version 22). A *P* value of <0.05 was considered statistically significant, and all *P* values were two-sided.

## 3. Results

### 3.1. Baseline Characteristics of Study Population

Table 2 lists the clinical characteristics of the MR patients with HF, aortic valve disease patients with HF, and control subjects without valve disease and HF. The HF status did not significantly differ between MR patients with HF and aortic valve disease patients with HF. The MR patients and the aortic valve disease patients did not significantly differ in age (*P* = 0.396), prevalence of hypertension (*P* = 0.710), prevalence of diabetes mellitus (*P* = 0.632), use of *β*-blockers (*P* = 1.000), or use of calcium channel blockers (*P* = 0.461). Left atrial size was significantly larger in the MR patients with HF compared to the aortic valve disease patients with HF (*P* = 0.019). However, left ventricular size and ejection fraction did not significantly differ between MR patients with HF and aortic valve disease patients with HF.

### 3.2. Comparison of Plasma Angiotensin II and Angiotensin 1~7 Concentrations among MR Patients with HF, Aortic Valve Disease Patients with HF, and Control Subjects without Valve Disease and HF

**Figure 1:**
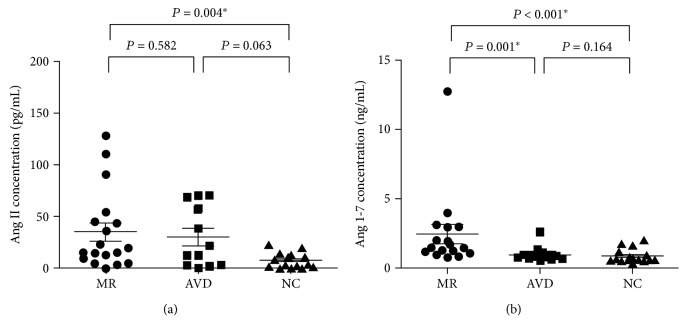
Plasma angiotensin II (Ang II) (a) and angiotensin 1~7 (Ang 1~7) (b) concentrations in mitral regurgitation (MR) patients with heart failure, in aortic valve disease (AVD) patients with heart failure, and in control subjects without valve disease or heart failure (NC). ^∗^*P* < 0.05.

Plasma angiotensin II concentrations did not significantly differ between MR patients and aortic valve disease patients (35.18 ± 9.03 vs. 30.13 ± 8.45 pg/mL, *P* = 0.582) (Figure 1(a)). However, MR patients had significantly higher plasma angiotensin II concentrations compared to controls (35.18 ± 9.03 vs. 7.94 ± 1.79 pg/mL, *P* = 0.004). Aortic valve disease patients had higher plasma angiotensin II concentrations compared to controls, but the difference did not reach statistical significance (30.13 ± 8.45 vs. 7.94 ± 1.79 pg/mL, *P* = 0.063).

The MR patients had significantly higher plasma angiotensin 1~7 concentrations compared to aortic valve disease patients (2.42 ± 0.68 vs. 0.96 ± 0.16 ng/mL, *P* = 0.001) (Figure 1(b)). The MR patients had significantly higher plasma angiotensin 1~7 concentrations compared to controls (2.42 ± 0.68 vs. 0.85 ± 0.12 ng/mL, *P* < 0.001). Plasma angiotensin 1~7 concentrations did not significantly differ between aortic valve disease patients and control subjects (0.96 ± 0.16 vs. 0.85 ± 0.12 ng/mL, *P* = 0.164).

### 3.3. Gene Expression in Renin-Angiotensin System Analyzed by Quantitative PCR in the Left Atrium: Comparisons of MR Patients with HF, Aortic Valve Disease Patients with HF, and Normal Controls

To determine the effects of MR and HF on the gene expression profiles of the renin-angiotensin system, gene expression profiles of the renin-angiotensin system in the left atrium were compared in left atrial tissues from MR patients with HF (*n* = 10), aortic valve disease patients with HF (*n* = 8), and normal controls (*n* = 6).  Table 1 shows the 17 genes examined in this study, all of which are known to have important roles in the KEGG pathway of the renin-angiotensin system (https://www.genome.jp/kegg-bin/show_pathway?hsa04614).

Table 3 shows that, compared to normal controls, the MR patients with HF had significantly downregulated expressions of angiotensin-converting enzyme (*ACE*) (fold change: 0.31, downregulation), angiotensin I converting enzyme (peptidyl-dipeptidase A) 2 (*ACE2*) (fold change: 0.15, downregulation), angiotensin II receptor, type 1 (*AT1*) (fold change: 0.51, downregulation), glutamyl aminopeptidase (*ENPEP*) (fold change: 0.66, downregulation), angiotensinogen (*AGT*) (fold change: 0.54, downregulation), cathepsin A (*CTSA*) (fold change: 0.33, downregulation), thimet oligopeptidase 1 (*THOP1*) (fold change: 0.44, downregulation), neurolysin, mitochondrial-like (*NLN*) (fold change: 0.32, downregulation), alanyl (membrane) aminopeptidase (*ANPEP*) (fold change: 0.15, downregulation), cathepsin G (*CTSG*) (fold change: 0.38, downregulation), leucyl/cystinyl aminopeptidase (*LNPEP*) (fold change: 0.24, downregulation), neprilysin (*MME*) (fold change: 0.19, downregulation), and carboxypeptidase A3 (*CPA3*) (fold change: 0.18, downregulation) in the left atrium. However, compared to normal controls, MR patients with HF had significantly upregulated expressions of *MAS1* oncogene (*MAS1*) (fold change: 3874.84, upregulation) and mineralocorticoid receptor (*NR3C2*) (fold change: 2.37, upregulation) in the left atrium (Table 3). The MR patients with HF also had upregulated expression of aldosterone synthase (*CYP11B2*) in the left atrium, which was not detected in normal controls (Table 3).

Compared to normal controls, the aortic valve disease patients had significantly downregulated expressions of *ACE* (fold change: 0.21, downregulation), *ACE2* (fold change: 0.03, downregulation), *AT1* (fold change: 0.32, downregulation), *ENPEP* (fold change: 0.44, downregulation), *THOP1* (fold change: 0.57, downregulation), *NLN* (fold change: 0.43, downregulation), *ANPEP* (fold change: 0.16, downregulation), *CTSG* (fold change: 0.37, downregulation), *LNPEP* (fold change: 0.57, downregulation), *MME* (fold change: 0.04, downregulation), and *CPA3* (fold change: 0.16, downregulation) in the left atrium. In contrast, the aortic valve disease patients with HF had significantly upregulated expression of *MAS1* (fold change: 292.81, upregulation) in the left atrium compared to normal controls (Table 3).

**Figure 2:**
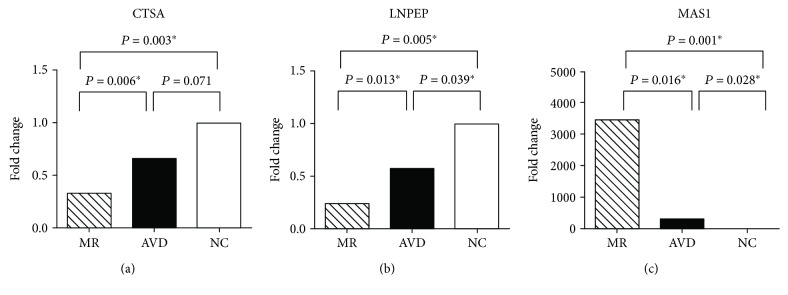
Quantitative determination of mRNA of (a) cathepsin A (*CTSA*), (b) leucyl/cystinyl aminopeptidase (*LNPEP*), and (c) *MAS1* oncogene (*MAS1*) by real-time RT-PCR in the left atrium in mitral regurgitation (MR) patients with heart failure (*n* = 10), in aortic valve disease patients (AVD) with heart failure (*n* = 8), and in purchased samples from normal subjects (NC) (*n* = 6). ^∗^*P* < 0.05.

Figure 1 shows that the MR patients with HF and the aortic valve disease patients with HF had similar plasma angiotensin II concentrations. However, Table 3 and Figure 2 show that, compared to the aortic valve disease patients with HF, the MR patients with HF had significantly downregulated expression of *CTSA* (fold change: 0.53, downregulation, *P* = 0.006 by univariate analysis (Figure 2); *P* = 0.004 with adjustment for plasma angiotensin II concentration and *P* = 0.004 with adjustment for left atrial size by analysis of covariance) and significantly downregulated expression of *LNPEP* (fold change: 0.48, downregulation, *P* = 0.013 by univariate analysis (Figure 2); *P* = 0.022 with adjustment for plasma angiotensin II concentration and *P* = 0.030 with adjustment for left atrial size by analysis of covariance) in the left atrium. In contrast, Figure 2 shows that, compared to the aortic valve disease patients with HF, the MR patients with HF had significantly upregulated expression of *MAS1* expression (fold change: 189.18, upregulation, *P* = 0.016 by univariate analysis (Figure 2); *P* = 0.003 with adjustment for plasma angiotensin II concentration and *P* = 0.007 with adjustment for left atrial size by analysis of covariance) in the left atrium. Expression of renin and angiotensin II receptor type 2 in the left atrium was not detected in most MR patients with HF and in most aortic valve disease patients with HF.

The MR patients with and without treatment with renin-angiotensin system blockers (*n* = 7 vs. *n* = 3) did not significantly differ in expressions of *ACE* (11.85 ± 0.53 vs. 13.07 ± 1.00, *P* = 0.305), *ACE2* (11.81 ± 0.68 vs. 10.90 ± 2.57, *P* = 0.770), *AT1* (13.58 ± 0.48 vs. 12.66 ± 0.30, *P* = 0.197), *ENPEP* (8.78 ± 0.18 vs. 9.02 ± 0.71, *P* = 0.606), *AGT* (5.20 ± 0.37 vs. 5.13 ± 0.48, *P* = 0.909), *CTSA* (6.44 ± 0.22 vs. 5.77 ± 0.65, *P* = 0.305), *THOP1* (9.75 ± 0.53 vs. 8.81 ± 0.65, *P* = 0.210), *ANPEP* (10.97 ± 0.60 vs. 8.96 ± 0.31, *P* = 0.143), *CTSG* (12.01 ± 0.60 vs. 10.94 ± 0.57, *P* = 0.245), *LNPEP* (8.43 ± 0.65 vs. 6.70 ± 0.80, *P* = 0.138), *MME* (11.33 ± 0.67 vs. 10.23 ± 0.21, *P* = 0.380), *CPA3* (11.89 ± 0.85 vs. 9.24 ± 0.92, *P* = 0.079), and *MAS1* (6.74 ± 1.21 vs. 5.46 ± 1.12, *P* = 0.569).

### 3.4. Tissue Angiotensin II and Angiotensin 1~7 Concentrations in the Left Atrium Compared between MR Patients with HF and Control Subjects without Valve Disease or HF

The MR patients (*n* = 12) had significantly lower tissue angiotensin II concentrations compared to control subjects (*n* = 5) without valve disease or HF (4.44 ± 1.04 vs. 9.60 ± 2.69 pg/mL, *P* = 0.023). Most of the MR patients in this study had received angiotensin-converting enzyme inhibitors or angiotensin II receptor blockers (Table 2). Angiotensin II receptor blockers have been shown to reduce tissue angiotensin II concentrations in the atria [7]. Tissue angiotensin 1~7 concentrations did not significantly differ between MR patients (*n* = 11) and control subjects (*n* = 5) without valve disease and HF (2.13 ± 0.32 vs. 2.08 ± 0.59 ng/mL, *P* = 0.865).

## 4. Discussion

This study showed that gene expression patterns of the renin-angiotensin system in the left atrium in MR patients with HF differed from those in aortic valve disease patients with HF and normal controls. Notably, for three genes in the renin-angiotensin system (*CTSA*, *LNPEP*, and *MAS1*), the differences were statistically significant.

To date, the gene expression profiles of the renin-angiotensin system in the atrial myocardium of MR patients have never been examined. A previous study using an atrial fibrillation pig model showed that atrial myocytes express all components of the renin-angiotensin system and undergo structural changes in response to rapid atrial pacing [8]. In another study, a canine model showed that an increased left ventricular mass was associated with increased *ACE* expression, increased chymase activity, and increased angiotensin II expression in the left ventricular myocardium [9]. In contrast, our study showed that *ACE* gene expression was decreased in the left atrial myocardium of MR patients when compared to normal controls. The different *ACE* expression in the left atrium and left ventricle in MR patients could be attributable to different hemodynamic stress on the left atrium and left ventricle. Renin-angiotensin system blockers might also have different effects on atrial and ventricular remodeling [10, 11].

Cathepsins, which are lysosomal proteases known to degrade unwanted intracellular or endocytosed proteins, reportedly play functional roles in the pathogenesis of heart disease by contributing to matrix turnover, chamber dilation, and structural remodeling [12]. Cathepsins may also be useful biomarkers for cardiovascular disease and pharmacological targets to remedy the progression of cardiovascular disease [12]. To date, however, information regarding the role of cathepsin A (*CTSA*) in cardiovascular disease is still limited. In vitro cathepsin A rapidly inactivates endothelin-1. Cathepsin A also hydrolyzes angiotensin I, which transforms angiotensin I into angiotensin-(1-9) or converts it to angiotensin II [13, 14]. Both angiotensin II and endothelin-1 have mitogenic effects on cardiac myocytes and play important roles in atrial structural remodeling in patients with structural heart disease and patients with atrial fibrillation [15, 16]. In the current study, cathepsin A expression in the left atrium was significantly downregulated in MR patients in comparison with aortic valve disease patients and normal controls. Therefore, increased tissue endothelin-1 levels in MR patients may contribute to atrial structural remodeling.

Leucyl/cystinyl aminopeptidase (*LNPEP*) is a catalyst in the final step of conversion of angiotensinogen to angiotensin IV. Leucyl/cystinyl aminopeptidase also cleaves vasopressin, oxytocin, bradykinin, and other peptide hormones [17]. Leucyl/cystinyl aminopeptidase is reportedly linked to glucose transport and utilization and to cognitive function [18, 19]. Interestingly, our prior study showed increased glycogen accumulation in the atrial myocytes of MR patients [5]. The MR patients in the current study showed significant downregulation of leucyl/cystinyl aminopeptidase expression in the left atrium in comparison with aortic valve disease patients and normal controls. Mice deficient in leucyl/cystinyl aminopeptidase reportedly have an increased heart size [20]. However, additional information regarding the role of leucyl/cystinyl aminopeptidase in human cardiovascular disease is limited.

The *MAS1* oncogene, a G-protein-coupled receptor and an endogenous receptor for the angiotensin-(1-7), can hetero-oligomerize with the AT1 receptor. By doing so, *MAS1* oncogene acts as a physiological antagonist to the AT1 receptor and activates the antiproliferative, antifibrotic, and antithrombotic effects of angiotensin-(1–7) [21, 22]. In the current study, *MAS1* expression in the left atrium was significantly upregulated in the MR patients in comparison with aortic valve disease patients and normal controls. In contrast, expression of AT1 receptor in the left atrium was significantly downregulated in the MR patients in comparison with normal controls. However, tissue angiotensin 1~7 concentrations did not significantly differ between MR patients and control subjects without valve disease and HF. The cardiac expression of *MAS* reportedly responds to various pathological stimuli, which suggests that *MAS* may be involved in homeostasis of the heart as well as the establishment and progression of cardiac disease [23].

Taken together, the data obtained in this study indicate that expressions of *CTSA*, *LNPEP*, and *MAS1* in the renin-angiotensin system in the left atrial myocardium of MR patients substantially differed from those in aortic valve disease patients, even after adjustment for plasma angiotensin II concentration and left atrial size. These differential gene expressions result from the biological response to the volume overload induced by MR and might have implications for atrial structural remodeling (hypertrophy, myolysis, dedifferentiation, apoptosis, and fibrosis) [4, 5, 7, 24] and atrial enlargement in MR patients.

### 4.1. Study Limitations

This study has several limitations. First, although significant differences were observed among the groups in this study, the number of subjects was relatively small. Therefore, studies with larger sample sizes are warranted. Second, most of the MR patients enrolled in the study had received renin-angiotensin system blockers, which may have modified the expressions of some genes. Notably, however, expressions of the 13 genes of the renin-angiotensin system did not significantly differ between MR patients treated with and without renin-angiotensin system blockers. Finally, this study did not specifically investigate the functional and regulatory roles of *CTSA*, *LNPEP*, and *MAS1* in atrial structural remodeling in MR patients.

## 5. Conclusions

Expressions of genes in the renin-angiotensin system, especially *CTSA*, *LNPEP*, and *MAS1*, in the left atrium in MR patients significantly differed from those in aortic valve disease patients and normal controls, and the differences were independent of circulating angiotensin II levels. The results of this study can provide a rationale for pharmacological therapies or posttranslational regulation therapies targeting differentially expressed genes in the renin-angiotensin system to remedy the structural remodeling associated with atrial enlargement and the progression of HF in patients with MR.

## Figures and Tables

**Table 1:** TaqMan real-time PCR assay identification.

Gene name	Assay identification
*ACE*	Hs00174179_m1
*ACE2*	Hs01085333_m1
*AT1*	Hs01096939_m1
*AT2*	Hs00169126_m1
*ENPEP*	Hs00989750_m1
*AGT*	Hs01586213_m1
*CTSA*	Hs00264902_m1
*THOP1*	Hs00162760_m1
*NLN*	Hs00252959_m1
*ANPEP*	Hs00174265_m1
*CTSG*	Hs0113415_g1
*LNPEP*	Hs00893646_m1
*MME*	Hs00153510_m1
*CMA1*	Hs00156558_m1
*MAS1*	Hs00267157_s1
*CPA3*	Hs00150019_m1
*Renin*	Hs00982555_m1
*GAPDH*	Hs03929097_g1

*ACE*: angiotensin-converting enzyme; *ACE2*: angiotensin I converting enzyme (peptidyl-dipeptidase A) 2; *AT1*: angiotensin II receptor, type 1; *AT2*: angiotensin II receptor, type 2; *ENPEP*: glutamyl aminopeptidase; *AGT*: angiotensinogen; *CTSA*: cathepsin A; *THOP1*: thimet oligopeptidase 1; *NLN*: neurolysin, mitochondrial-like; *ANPEP*: alanyl (membrane) aminopeptidase; *CTSG*: cathepsin G; LNPEP: leucyl/cystinyl aminopeptidase; *MME*: neprilysin; *CMA1*: mast cell protease 2-like; *MAS1*: *MAS1* oncogene; *CPA3*: carboxypeptidase A3; PCR: polymerase chain reaction.

**Table 2:** Baseline clinical characteristics of the study subjects.

	MR (*n* = 18)	AVD (*n* = 12)	NC (*n* = 16)	*P* value
Age (years)	57 ± 11	60 ± 12	49 ± 12	0.053
Male (%)	5 (27.8%)	9 (75.0%)	10 (62.5%)	0.024
Smoking (%)	2 (5.6%)	1 (8.3%)	2 (12.5%)	0.772
Body mass index (kg/m^2^)	23.7 ± 2.6	25.3 ± 3.4	23.6 ± 3.4	0.168
Hypertension (%)	8 (44.4%)	7 (58.3%)	0 (0.0%)	0.002
Diabetes mellitus (%)	3 (16.7%)	1 (8.3%)	0 (0.0%)	0.227
Hyperlipidemia (%)	6 (33.3%)	3 (25.0%)	NA	0.704^a^
NYHA				0.324^a^
Functional class I (%)	2 (11.1%)	3 (25.0%)		
Functional class II (%)	7 (38.9%)	5 (41.7%)		
Functional class III (%)	9 (50.0%)	3 (25.0%)		
Functional class IV (%)	0 (0.0%)	1 (8.3%)		
Aortic valve disease (%)	0 (0.0%)	12 (100.0%)		
Tricuspid regurgitation (%)	7 (38.9%)	1 (8.3%)		0.099^a^
*β*-Blockers (%)	3 (16.7%)	2 (16.7%)		1.000^a^
Calcium channel blockers (%)	5 (27.8%)	5 (41.7%)		0.461^a^
Angiotensin-converting enzyme inhibitors or angiotensin II receptor blockers (%)	14 (77.8%)	4 (33.3%)		0.024^a^
Systolic blood pressure (mmHg)	127.3 ± 21.7	131.5 ± 17.0	124.3 ± 12.3	0.672
Diastolic blood pressure (mmHg)	77.8 ± 11.2	71.6 ± 7.7	75.6 ± 8.7	0.364
Heart rate (beats/min)	78.2 ± 12.6	71.1 ± 10.6	78.9 ± 9.1	0.162
Creatinine (mg/dL)	0.9 ± 0.6	0.9 ± 0.2	0.8 ± 0.2	0.089
eGFR (mL/min/1.73 m2)	86.2 ± 22.3	79.0 ± 17.3	92.4 ± 17.1	0.093
White blood cell count (10^3^/*μ*L)	6.1 ± 1.7	5.8 ± 1.5	6.0 ± 1.4	0.775
Left atrial diameter (mm)	43.7 ± 5.1	38.8 ± 4.7	NA	0.019^a^
Left atrial maximal volume (mL)	86.4 ± 38.8	58.8 ± 38.0	NA	0.083^a^
Left atrial ejection fraction (%)	51.6 ± 14.4	44.7 ± 19.5	NA	0.376^a^
Left ventricular end-diastolic diameter (mm)	56.3 ± 6.4	58.3 ± 11.9	NA	0.384^a^
Left ventricular ejection fraction (%)	67.7 ± 11.0	62.2 ± 12.9	NA	0.396^a^

Data are presented as mean ± SD or number (percentage); AVD: aortic valve disease; MR: mitral regurgitation; NC: control subjects without valve disease and heart failure; NYHA: New York Heart Association; ^a^*P* value: MR vs. AVD.

**Table 3:** Comparison of mRNA levels through quantitative PCR in the left atria among MR patients with heart failure, aortic valve disease patients with heart failure, and normal controls.

Gene name	MR (*n* = 10)	AVD (*n* = 8)	NC (*n* = 6)	*P* value		
				MR vs. NC	AVD vs. NC	MR vs. AVD
*ACE*	12.22 ± 0.48	12.37 ± 0.31	9.85 ± 0.66	0.023	0.005	0.657
*ACE2*	11.61 ± 0.69	12.83 ± 0.34	7.57 ± 0.32	0.003	0.002	0.149
*AT1*	13.27 ± 0.36	14.05 ± 0.47	12.04 ± 0.29	0.013	0.010	0.126
*ENPEP*	8.86 ± 0.24	9.52 ± 0.31	8.14 ± 0.10	0.007	0.002	0.068
*AGT*	5.18 ± 0.28	4.91 ± 0.25	4.09 ± 0.22	0.007	0.053	0.594
*CTSA*	6.24 ± 0.25	5.13 ± 0.19	4.44 ± 0.31	0.003	0.071	0.006
*THOP1*	9.47 ± 0.42	8.83 ± 0.24	7.89 ± 0.08	0.002	0.007	0.424
*NLN*	10.60 ± 0.29	10.00 ± 0.18	8.72 ± 0.30	0.005	0.020	0.214
*ANPEP*	10.52 ± 0.55	10.66 ± 0.73	7.03 ± 0.52	0.001	0.005	0.923
*CTSG*	11.71 ± 0.48	11.73 ± 0.47	9.94 ± 0.59	0.042	0.042	0.949
*LNPEP*	7.91 ± 0.55	6.17 ± 0.27	5.18 ± 0.40	0.005	0.039	0.013
*MME*	11.08 ± 0.54	10.66 ± 0.23	7.88 ± 0.66	0.010	0.002	0.441
*CMA1*	13.88 ± 0.46	13.43 ± 0.38	11.50 ± 0.94	0.063	0.053	0.298
*CPA3*	11.30 ± 0.77	10.59 ± 0.41	7.64 ± 0.71	0.007	0.014	0.441
*MAS1*	6.35 ± 0.90	12.19 ± 1.44	16.39 ± 0.69	0.001	0.028	0.016
*NR3C2*	4.89 ± 0.14		6.08 ± 0.31	0.007		
*CYP11B2*	4.56 ± 0.28		Unmeasurable			

Data are presented as mean ± SEM; quantitative RT-PCR values are presented in △Cq units; MR: mitral regurgitation; AVD: aortic valve disease; NC: purchased samples from normal subjects. *ACE*: angiotensin-converting enzyme; *ACE2*: angiotensin I converting enzyme (peptidyl-dipeptidase A) 2; *AT1*: angiotensin II receptor, type 1; *ENPEP*: glutamyl aminopeptidase; *AGT*: angiotensinogen; *CTSA*: cathepsin A; *THOP1*: thimet oligopeptidase 1; *NLN*: neurolysin, mitochondrial-like; *ANPEP*: alanyl (membrane) aminopeptidase; *CTSG*: cathepsin G; *LNPEP*: leucyl/cystinyl aminopeptidase; *MME*: neprilysin; *CMA1*: mast cell protease 2-like; *CPA3*: carboxypeptidase A3; *MAS1*: *MAS1* oncogene; *NR3C2*: mineralocorticoid receptor; *CYP11B2*: aldosterone synthase gene.

## Data Availability

The data used to support the findings of this study are included within the article.
